# Out-of-hospital cardiac arrest: determinant factors for immediate survival after cardiopulmonary resuscitation^1^

**DOI:** 10.1590/0104-1169.3453.2452

**Published:** 2014

**Authors:** Daniela Aparecida Morais, Daclé Vilma Carvalho, Allana dos Reis Correa

**Affiliations:** 2 PhD, RN, Serviço de Atendimento Móvel de Urgência, Belo Horizonte, MG, Brazil; 3 PhD, Associate Professor, Escola de Enfermagem, Universidade Federal de Minas Gerais, Belo Horizonte, MG, Brazil; 4 PhD, Adjunct Professor, Escola de Enfermagem, Universidade Federal de Minas Gerais, Belo Horizonte, MG, Brazil

**Keywords:** Out-of-Hospital Cardiac Arrest, Cardiopulmonary Resuscitation, Emergency Medical Services, Ambulances, Prehospital Care

## Abstract

**OBJECTIVE:**

to analyze determinant factors for the immediate survival of persons who receive cardiopulmonary resuscitation from the advanced support units of the Mobile Emergency Medical Services (SAMU) of Belo Horizonte.

**METHOD:**

this is a retrospective, epidemiological study which analyzed 1,165 assistance forms, from the period 2008 - 2010. The collected data followed the Utstein style, being submitted to descriptive and analytical statistics with tests with levels of significance of 5%.

**RESULTS:**

the majority were male, the median age was 64 years, and the ambulance response time, nine minutes. Immediate survival was observed in 239 persons. An association was ascertained of this outcome with "cardiac arrest witnessed by persons trained in basic life support" (OR=3.49; p<0.05; CI 95%), "cardiac arrest witnessed by Mobile Emergency Medical Services teams" (OR=2.99; p<0.05; CI95%), "only the carry out of basic life support" (OR=0.142; p<0.05; CI95%), and "initial cardiac rhythm of asystole" (OR=0.33; p<0.05; CI 95%).

**CONCLUSION:**

early access to cardiopulmonary resuscitation was related to a favorable outcome, and the non-undertaking of advanced support, and asystole, were associated with worse outcomes. Basic and advanced life support techniques can alter survival in the event of cardiac arrest.

## Introduction

Currently, the cardiovascular diseases constitute the most important group of causes of death in Brazil. Among these, the ischemic heart diseases stand out as the principal causes of cardiac arrest (CA)^(^ 1 ^)^.

For the assistance given to CA victims to be effective, some actions are necessary, such as the early recognition of the situation, the rapid activation of the emergency medical system, and the swift undertaking of cardiopulmonary resuscitation (CPR)^(^ 2 ^)^.

In Brazil, there was a need for the organization of a regionalized and hierarchized emergency care network, which led to the implementation of the Mobile Emergency Medical Services (SAMU, in Portuguese) in 2003^(^ 3 ^)^.

The SAMU of Belo Horizonte (BH) attends many cases of CA, according to a study undertaken in 2005, which ascertained that this service's advanced support units (ASU) attended approximately 5,058 occurrences, and that 30% of these were people with CA^(^ 3 ^)^.

Taking into account the panorama of morbidity and mortality of the cardiovascular diseases, associated with the high number of attendances made by the SAMU-BH for people with CA, this study was undertaken, with the objective of analyzing determinant factors for the immediate survival of people who received cardiopulmonary resuscitation from the Advanced Support teams of the Mobile Emergency Medical Services of Belo Horizonte.

## Method

This is an observational, retrospective study undertaken based on the identification and analysis of 1,165 records of pre-hospital attendances of people aged over 18 years old, victims of CA with a probable cardiac origin, who received CPR in an out-of-hospital environment, attended by the ASU teams of SAMU-BH, in the period between 01/01/2008 (the effective implementation of the automated external defibrillator in the basic support units of SAMU-BH) and 10/17/2010 (changes in the Guidelines for Cardiopulmonary Resuscitation and Emergency Cardiovascular Care, published in 10/18/2010). These were selected based on the manual manipulation of 27,619 files referent to all of the attendances made by the ASU teams in the above-mentioned period. The flowchart for inclusion and determination of the population is presented in Figure 1. 

The data collection instrument was constructed based on the Utstein style^(^ 4 ^)^, a guide for international use, already translated and validated in the Portuguese language^(^ 5 ^)^, created to guide and standardize studies in the area of CA and thus to allow comparisons to be made between the studies undertaken. The hospital records from which the data were compiled were not formatted with all the variables determined by the Utstein style^(^ 4 ^)^, with: sex, age, response time (time between the call to Emergency Medicine Service (EMS) until the its arrival on site), witnessed CA, first monitored cardiac rhythm, the CPR attempted prior to the arrival of the advanced support teams, defibrillation, drugs and the return of spontaneous circulation (ROSC) being the variables used in the original instrument. The category of "not recorded" (NR) was created for all the variables. 

The data were collected by the researchers themselves and were later subjected to statistical analysis (descriptive analysis and logistic regression) using the R software, version 2.15.0. Non-conditional analysis (univariate) was undertaken with the level of p<0.250 in order to undertake the multivariate phase which considered p<0.050, used the stepwise method and selected the following variables: sex, age, ambulance response time, witnessed CA, CPR initiated prior to the arrival of the advanced support teams, the initial cardiac rhythm, the type of intervention, defibrillation, and the use of epinephrine, atropine sulfate, and amiodarone hydrochloride. The explained variable (Y) was whether or not immediate survival followed the CPR, being characterized by the evidence of a palpable pulse or measurable blood pressure in the patient following CPR, in a pre-hospital environment, until the patient's admission in a hospital unit^(^ 4 ^)^.

This study's research project was approved by the Ethics Committee of the Federal University of Minas Gerais (Opinion N. 035.0.410.209-9).

## Results

Of the sample studied, 58.9% of the people were male. Nearly all the records (98.6%) recorded age, which varied from 18 to 103 years old. The median age was 64 years (SD=16.6), with 75.0% of the people being aged up to 76 years old.

Comorbidities were recorded in 37.4% of the records, and of these, in 37.6%, there were records of two or more comorbidities. The highest percentage was for systemic arterial hypertension (18.4%), followed by heart disease (15.8%), diabetes mellitus (8.8%), smoking (2.7%), and alcoholism (3.7%).

In relation to the ambulance response time, it was ascertained that the record was present in 70.6% of the records and that it varied from 1 to 69.0 minutes; the median was nine minutes (SD=6.0), and in at least 75.0% of the occasions it was below or equal to 13.0 minutes.

The characterization of the data on the period of call-out of the ambulance, witnessed CA, CPR prior to the arrival of the ASU, the first monitored cardiac rhythm evaluated by the ASU teams and defibrillation is presented in Table 1.

**Table 1:** Distribution of the characteristics of the attendances to people who received cardiopulmonary resuscitation from the teams of the Advanced Support Units of the Mobile Emergency Medical Service of Belo Horizonte, by period of call-out for Advanced Support Unit, witnessed cardiac arrest prior to the arrival of the Advanced Support Unit, cardiopulmonary resuscitation prior to the arrival of the Advanced Support Unit, first monitored cardiac rhythm, and defibrillation. Belo Horizonte, MG, Brazil, 2008-2010

Variable			n	%
Period of call-out of Advanced Support Unit				
	Morning (06:00-11:59)		342	37.1
	Afternoon (12:00-17:59)		253	27.5
	Night-time (18:00-23:59)		217	23.6
	Very early morning (00:00-05:59)		109	11.8
	Not recorded		244	20.9
Witnessed cardiac arrest prior to the arrival of the Advanced Support Unit				
	Yes		161	29.1
		Layperson	65	40.3
		Teams of the Mobile Emergency Medical Services	73	45.4
		Persons trained in basic life support	23	14.3
	No		392	70.9
	Not recorded		612	52,5
Cardiopulmonary resuscitation prior to the arrival of the Advanced Support Unit				
	Yes		565	66
		Layperson	19	3.4
		Persons trained in basic life support	546	96.6
	No		291	34
	Not recorded		309	26.5
First monitored cardiac rhythm				
	Ventricular fibrillation/Pulseless ventricular tachycardia		225	32.2
	Pulseless electrical activity (PEA)		124	17.7
	Asystole		350	50.1
	Not recorded		466	40
Defibrillation				
	Yes		443	44.1
		Automated External Defibrillator	103	23.3
		Manual Defibrillator	188	42.4
		Automated External Defibrillator and Manual Defibrillator	152	34.3
	No		561	55.9
	Not recorded		161	13.8

Source: Mobile Emergency Medical Service, Belo Horizonte, MG, Brazil

It stands out that in a considerable percentage of files (40.0%) the first monitored cardiac rhythm detected by the advanced support teams was not recorded. 

In relation to the use of drugs, epinephrine was administered to 68.7% of the people during CPR, followed by atropine sulfate (55.9%) and amiodarone hydrochloride (21.4%). Medications such as vasopressin, lidocaine, sodium bicarbonate, calcium gluconate, and magnesium sulfate, used with a lower frequency, were grouped in a variable determined "other medications" and which corresponded to 6.9% of the cases. 

Regarding the outcome of the assistance, in the pre-hospital environment, the majority of the persons (78.1%) died. 

Table 2 presents the complete model, after the stepwise method, with the variables selected to make up the multivariate model. 

**Table 2:** Multivariate model for persons who received Cardiopulmonary Resuscitation from the teams of the advanced support units of the Mobile Emergency Medical Service of Belo Horizonte, according to immediate survival. Belo Horizonte, MG, Brazil, 2008-2010

Variables (n=1,041)		OR	p-value	CI 95%	
Cardiac Arrest witnessed					
	By persons trained in basic life support	3.495	0.007	1.409	8.670
	By teams from the advanced support or basic support units	2.998	0.000	1.683	5.340
Type of intervention					
	Basic Life Support	0.142	0.000	0.056	0.361
First monitored Cardiac rhythm					
	Pulseless Electrical Activity (PEA)	0.616	0.056	0.375	1.013
	Asystole	0.339	0.000	0.220	0.524

Source: Mobile Emergency Medical Service, Belo Horizonte, MG, Brazil

In accordance with Table 1, it may be ascertained that in the situations in which the CA was witnessed by persons trained in basic life support (OR=3.5 p<0.05) or by teams from the SAMU (OR=3.0 p<0.05) there is a greater likelihood of immediate survival. However, when only basic life support was undertaken (OR=0.14 p<0.05) or when the patient's initial cardiac rhythm detected by the advanced support teams was asystole (OR=0.4 p<0.05) this chance was lower.

## Discussion

It was verified that the occurrence of CA in males was 1.5 times greater than in females. In Brazil, four studies on the occurrence of CA in the pre-hospital environment - two in Belo Horizonte/Minas Gerais^(^ 3 ^,^ 6 ^)^, one in Araras/São Paulo^(^ 7 ^)^ and another in Porto Alegre/Rio Grande do Sul^(^ 8 ^)^ have reported the occurrence of CA as approximately two times greater in men. 

The median of 64 years of age found in this study is in line with studies undertaken in two SAMU in Brazil, in which medians of 63 and 66 years of age respectively were found^(^ 7 ^-^ 8 ^)^.

In relation to the morbid antecedents, it was ascertained that of the files in which this information is recorded, systemic arterial hypertension, the cardiac diseases and diabetes mellitus were the most prevalent. However, the majority of the patients who received CPR had no comorbidities reported, which does not mean that they had none, as family members or persons present at the time of the attendance might not have known of the existence of the patient's health problems.

In the study undertaken in Araras/São Paulo, the comorbidities mentioned above were also the most prevalent; however, the most prevalent was heart disease followed by systemic arterial hypertension and diabetes mellitus^(^ 7 ^)^.

The ambulance response time, that is, the time which passed in minutes between the transmission of the call to the team by the Dispatch Center until the arrival of the ambulance in the specified place is one of the performance indicators for a pre-hospital care service.

In this study, the median response time was nine minutes. In comparing this variable with studies undertaken previously in the same service, it may be perceived that there was a reduction in the time spent travelling, which, in 2007^(^ 3 ^)^ was approximately 10.3 minutes, and was 10.4 minutes in 2010^(^ 6 ^)^. One explanation may be the increase of the fleet and of advanced support equipment for care, which from 2007 to the time of the study increased by three, to six units. The ambulances' response times would probably have been even lower if, in the last few years, the main streets of the city of Belo Horizonte had not been undergoing major rebuilding so that the city could host major sporting events of an international scope. The majority of the events attended by the advanced support teams were in the morning period, suggesting that CA occurs also in this period. 

Studies report the relation of the period of the day to the possibility of occurrences of CA. Authors have verified that there is a greater risk of a person having a CA up to three hours after waking up than in the other hours of the day. This is due to an increase in blood pressure and cardiac frequency, which raises the muscle tone, the viscosity of the blood and the platelet aggregation^(^ 9 ^-^ 10 ^)^.

The CA was witnessed by somebody (29.1%), in the majority of cases this being by lay persons. A similar situation was found in the studies undertaken in Araras/São Paulo^(^ 7 ^)^ and Porto Alegre/Rio Grande do Sul^(^ 8 ^)^, in which the authors reported 35.17% and 28.0%, respectively.

CPR was undertaken prior to the arrival of the advanced support teams, by members of the basic support teams, who are people trained in basic life support, and to a lesser degree by lay persons.

It is perceived that although the doctors of this service provide telephone guidance for people on how to proceed until the ambulance arrives, this guidance is not always followed. Emotional imbalance due to the situation, lack of appropriate skill for undertaking CPR, and the possibility of the victim being a close relative often stop lay persons from functioning appropriately^(^ 6 ^)^.

It is extremely important to train people to be able to act when faced with a CA, as undertaking CPR, until the emergency medical service arrives, can increase the victim's chances of survival^(^ 11 ^)^.

Ventricular fibrillation (VF) and ventricular tachycardia without a pulse (VT), frequently, are the rhythms found in persons with witnessed CA, as a result of which it is extremely important that both CPR and defibrillation should be undertaken at an early stage. The chance of survival reduces by 7.0%-10.0% with each minute of delay in defibrillation, and pulseless VF/VT can deteriorate to asystole as time passes, but undertaking CPR can prolong pulseless VF/VT, thus increasing the chances of successful defibrillation^(^ 12 ^)^.

The advanced support teams verify the cardiac rhythm of the victim of CA during the first approach on the scene. This rhythm is not necessarily the same as the initial rhythm in the CA, given that it is verified some minutes after the CA occurs, except in those cases of CA witnessed by this team. 

The use of epinephrine, atropine sulfate and amiodarone hydrochloride was reported; these are medications commonly used in assisting persons with CA. Epinephrine is generally used in all the CA rhythms; amiodarone, an antiarrhythmic, is only used in those situations in which pulseless VF/VT does not respond to the electric shock^(^ 1 ^,^ 13 ^)^.

It is important to report that with the publicizing of the Guidelines for Cardiopulmonary Resuscitation and Emergency Cardiovascular Care in October 2010, atropine stopped being the drug of choice for attending CA^(^ 13 ^)^. At the time of this study, however, it was used for non-shockable rhythms (PEA <60bpm and asystole)^(^ 14 ^)^.

It is noted that the percentage of immediate survival of the victims in this study was lower than that identified (25.1%) in 2005^(^ 3 ^)^ in the same municipality. However, it was similar to that found (20.0%) in the study undertaken in Porto Alegre^(^ 8 ^)^.

The most critical factor for patients with CA is probably the time which passes between the start of the collapse and the beginning of the treatment, and the chance of survival is lower if this event is not witnessed by anybody.

In this study, it was ascertained that the people who had a CA which was witnessed by somebody trained in basic life support, or by some member of the SAMU team had a chance of immediate survival which was 3.5 and 2.9 times higher, respectively, when compared with people whose CA was not witnessed by these groups. However, the chance of immediate survival in people who received only basic life support, or in whom the cardiac rhythm of asystole was detected by the advanced support teams, was respectively 7.0 and 3.0 times lower, when compared with those persons who received - in addition to basic support - advanced life support, or with those people who had pulseless VF/VT as their initial cardiac rhythm. These findings are in consonance with the study also undertaken in the SAMU/BH, in 2005, which showed an association between immediate survival, CA witnessed by the care teams (OR=2.8 p<0.05), and asystole as the initial rhythm (OR=0.4 p<0.05)^(^ 3 ^)^.

Asystole is frequently considered a rhythm which confirms death, rather than an arrhythmia to be treated, as the results for survival are bleak^(^ 15 ^)^.

It is important to emphasize that immediate survival on its own does not imply the patient's recovery, as there are other factors which also influence the outcome^(^ 16 ^)^.

The principal limitations of this study are as follows: the search for data after the occurrence of the events, and access only to the information described in the attendance forms - which may have contributed to the absence of accurate information on important variables such as: the exact place where the CA occurred (at home, public highway, or other), the time when help was requested by telephone, the duration of the resuscitation, the time between collapse and defibrillation, and the interval between CA and the administration of the first dose of epinephrine. The absence of records of data whose recording is mandatory according to service protocols, such as the attendance times, the time when the team was actioned, the time it arrived, and the patient's progression and destination all influenced the presentation of the results and the statistical analysis. 

## Conclusion

This study identified some factors associated with immediate survival. It stands out that CA witnessed by a person trained in basic life support or by the care team is favorable to the outcome; while "the undertaking of basic life support alone" and "asystole as the initial rhythm" are unfavorable. 

These findings confirm the importance of having a well-organized pre-hospital care service, structured with trained teams, as well as the need for training and providing guidance to the lay population regarding how to proceed when faced with a person with CA. 

Cardiac arrest is the most serious clinical emergency and has the worst prognosis; however, it can be a transitory stage, and is reversible, with the possibility for patients to recover and return to their activities. For this, it is important for studies on late survival (discharge from hospital) and long-term survival (following discharge) to be undertaken. 
